# A Child with Lung Hypoplasia, Congenital Heart Disease, Hemifacial Microsomia, and Inguinal Hernia: Ipsilateral Congenital Malformations

**DOI:** 10.1155/2015/741540

**Published:** 2015-07-29

**Authors:** Chengming Fan, Can Huang, Jijia Liu, Jinfu Yang

**Affiliations:** Department of the Cardiothoracic Surgery, The Second Xiangya Hospital, Central South University, Middle Renmin Road 139, Changsha 410011, China

## Abstract

A 3-year-old Chinese boy was diagnosed with ipsilateral congenital malformations: right lung hypoplasia, dextroversion of heart, atrial septal defect, hepatic vein drainage directly into the right atrium, facial asymmetry, right microtia and congenital deafness, and indirect inguinal hernia. He underwent indirect inguinal hernia repair at the age of 2. Although without any facial plastic surgery performed, he underwent a repair of atrial septal defect and recovered uneventfully. At 6-month follow-up, the patient was free from any symptom of dyspnea; his heart function returned to the first grade.

## 1. Introduction

Lung hypoplasia or agenesis is part of the spectrum of malformations featured by incomplete development of lung tissue and is often associated with other ipsilateral congenital malformations [1]. Congenital malformations associated with pulmonary hypoplasia may be present in any system including cardiovascular system [2], gastrointestinal system, central nervous system, and musculoskeletal system. Here we described a 3-year-old Chinese boy, who was referred to us for heart murmur in right hemithorax. Subsequently, he was found to have hypoplasia of right lung, dextroversion of heart, atrial septal defect (ASD), hepatic vein drainage directly into the right atrium through coronary sinus, facial asymmetry, microtia, and indirect inguinal hernia.

## 2. Case Report

**Figure 1:**
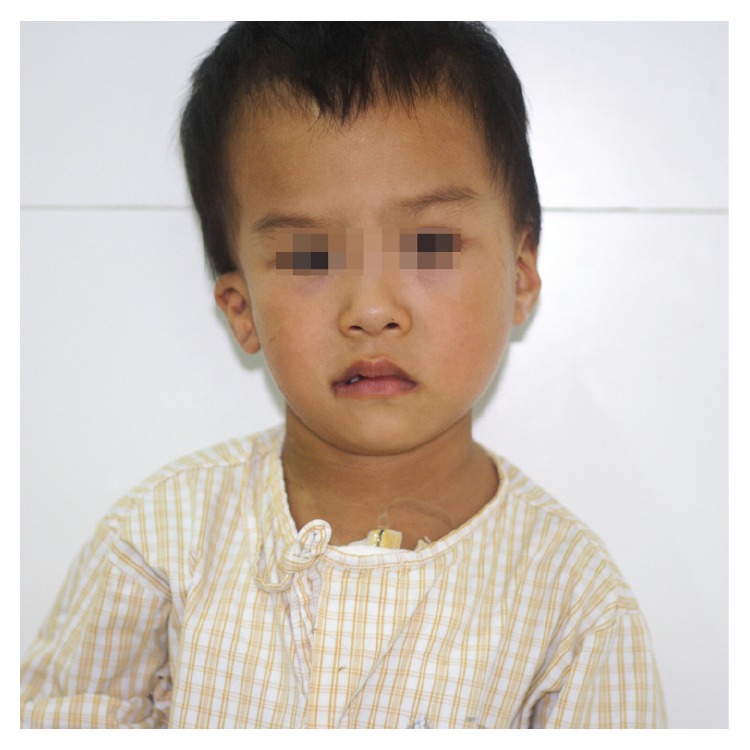
Frontal facial view showing facial asymmetry affecting the right side including microtia and downslanting zygomatic arch, jaw bone, mouth, and lips.

**Figure 2:**
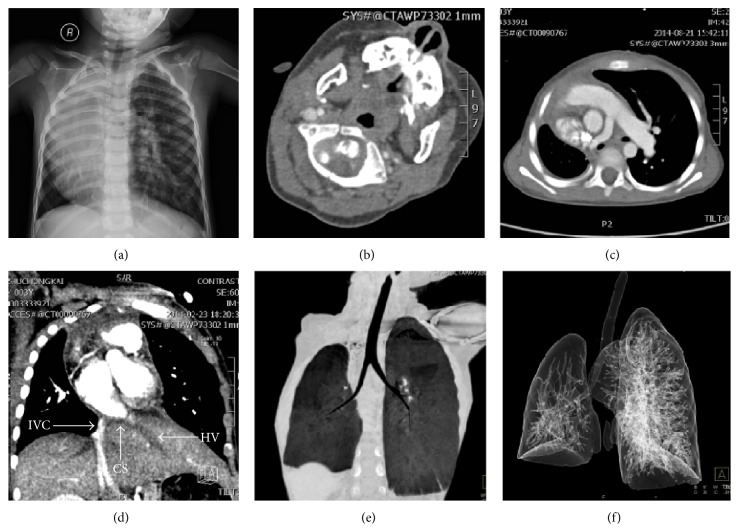
X-ray chest showing (a) a homogenous opacity occupying most of right hemithorax and compensatory hyperinflation on left side. Cranial computed tomography demonstrating (b) imperforation of right external acoustic meatus. The CECT chest in mediastinal window showing (c) right lung hypoplasia, left lung tissue crossing through the mediastinum, and hypoplastic right pulmonary artery and (d) hepatic vein that directly drains into right atrium thorough coronary sinus. CECT chest in lung window showing that (e) the right side of thoracic cage is mildly collapsed and no obvious branches of the right main bronchus could be observed. Volume-rendering computed tomography 3-dimensional reconstruction showing (f) right lung hypoplasia and a compensatory hyperplasia left lung.

A 3-year-old Chinese boy accompanied by his parents visited our outpatient clinic for a heart murmur detected at the age of 2 months. He lost hearing of his right ear from birth. He had no history of chest pain or heaviness of chest, wheeze, and dyspnea. There was no history of consanguineous marriage in their family. His birth history and perinatal period were uneventful. Physical examination showed facial asymmetry (face and mouth deviated to right side), right microtia with aural atresia (Figure 1), a surgical scar of right indirect inguinal hernia repair, stony dullness, and absent breath sounds in the right up chest. There was grade 3/6 systolic murmur on the right parasternal border under the second rib. A chest roentgenogram showed homogeneous opacity occupying the entire right up hemithorax, hyperinflated left lung, and mediastinal shift to the right (Figure 2(a)). A 12-lead electrocardiogram showed sinus tachycardia. Echocardiogram showed dextroversion of heart and a 17 mm sized ostium secundum defect, moderate pulmonary hypertension (MPAP = 40 mmHg). Computerized tomography (CT) of the brain and chest showed a blind-ending right external acoustic meatus (Figure 2(b)), the whole heart located in the right chest, and severe right lung hypoplasia with left lung tissue compensatory hyperplasia and crossing through the mediastinum. The transverse diameter of right pulmonary artery was 7 mm without evidence of right ascending pulmonary artery. Left pulmonary artery was compensatory broadening with a diameter of 14 mm (Figure 2(c)). Hepatic vein was draining directly into the right atrium through coronary sinus (Figure 2(d)). The right side of thoracic cage was mildly collapsed, and no obvious branches of the right main bronchus could be observed (Figures 2(e) and 2(f)). Color Doppler ultrasound examination of the abdomen and urinary system was all normal. He was diagnosed with ipsilateral congenital malformations: right lung hypoplasia, dextroversion of heart, ASD, hepatic vein drainage directly into the right atrium, facial asymmetry, right microtia with deafness, and indirect inguinal hernia. The patient underwent cardiac surgery of ASD repair 6 months after the hernioplasty of indirect inguinal hernia. Surgery progressed smoothly and no anomalous pulmonary venous connection was found. The patient was weaned from the ventilator 5 hours after surgery and discharged uneventfully on postoperative day 7. At 6-month follow-up, the patient was in good condition without the symptoms of dyspnea and palpitation. His heart function was NYHA class 1.

## 3. Discussion

Pulmonary agenesis is a rare malformation, which has been described as an isolated lesion or associated with other anomalies. Cardiac anomalies with pulmonary hypoplasia mainly include Ebstein's anomaly, tetralogy of Fallot (TOF), scimitar syndrome, pulmonary stenosis, and hypoplastic right heart [3, 4]. The cause of these ipsilateral congenital malformations remains unclear. Clinical presentation and the degree of respiratory compromise depend on the associated anomalies and the severity of hypoplasia. By far, there have been no effective treatment methods for pulmonary hypoplasia. In 2005, Festa et al. [2] successfully operated with a repair of total anomalous pulmonary venous connection (TAPVC) and modified Glenn anastomosis for a patient diagnosed with right lung hypoplasia associated with TOF and TAPVC. Patnaik et al. [5] reported a case of left lung hypoplasia associated with congenital pulmonary artery aneurysm and ventricular septal defect (VSD) in 2013. In order to reduce operative risks. The aneurysmal repair was performed first. The patient was discharged in stable condition leaving the VSD unclosed.

The hypoplastic right lung reported in this paper is associated with congenital heart malformation, right indirect inguinal hernia, right hemifacial microsomia, and right microtia with aural atresia. The congenital heart malformations are dextroversion of heart, ASD, and hepatic vein drainage directly into the right atrium through coronary sinus. In the literature there is no description of such ipsilateral congenital malformations. Earlier treatment of cardiac malformation and timely correction of craniofacial asymmetry and aural atresia for these congenital malformations were recommended.
